# An experimental study on the impact of prosthesis temperature on the biomechanical properties of bone cement fixation

**DOI:** 10.1186/s12893-023-02079-3

**Published:** 2023-07-05

**Authors:** Wanzhuo Chen, Haining Zhang

**Affiliations:** 1 Department of Joint Surgery, the Affiliated Hospital of Qingdao University, Qingdao, 266000 China; 2 State Key Discipline: Joint Surgery, The Affiliated Hospital of Qingdao University, Qingdao, 266000 Shandong Province China

**Keywords:** Total knee arthroplasty, Bone cement, Push-out force, Fixation strength

## Abstract

**Purpose:**

To investigate the effect of the femoral component and tibial plateau component temperature on the strength of cement fixation during total knee arthroplasty (TKA).

**Methods:**

Femoral prosthesis, tibial plateau prosthesis, and polypropylene mold base were used to simulate TKA for bone cement fixation. Pre-cooling or pre-warming of femoral and tibial plateau components at different temperatures (4 °C, 15 °C, 25 °C, 37 °C, 45 °C), followed by mixing and stirring of bone cement at laboratory room temperature (22 °C), were performed during research. The prosthesis and the base adhered together, and the bone cement was solidified for 24 h at a constant temperature of 37 °C to verify the hardness of the bone cement with a push-out test.

**Results:**

The push-out force of the femoral prosthesis after fixation was higher than that of the tibial plateau prosthesis, and with the increase of the prosthesis temperature, the push-out force after fixation of the bone cement also increased linearly and the porosity of the prosthetic cement in the tibia and femur decreased as the temperature increased.

**Conclusion:**

Without changing the mixing temperature and solidification temperature, the fixation strength of the femoral prosthesis is higher than that of the tibial plateau prosthesis. Properly increasing the temperature of the prosthesis can increase the push-out force of the fixation strength.

## Introduction

Total knee arthroplasty (TKA) is one of the most common procedures used to relieve joint pain in patients with end-stage osteoarthritis or rheumatoid arthritis of the knee. The demand for joint replacement has increased dramatically in recent years because of its favorable clinical results [1–3]. Likewise, other types of joint replacement surgery, including shoulder and elbow, are on the rise [4–6]. But at the same time, complications and all causes of readmission for knee replacement surgery are increasing [7]. Two main reasons for implant failure are aseptic loosening and infection. Aseptic loosening can originate from a variety of sources, these include fretting of the implant relative to the bone during loading, generation of implant wear particles that lead to inflammation and bone resorption, and poor osseointegration between implant and bone [8].

Bone cement is mainly supplied as a two-component system consisting of powder polymethyl methacrylate (PMMA) copolymer and liquid methyl methacrylate (MMA) monomer. The polymerization process is divided into four stages: mixing, waiting, working, and hardening. The hardening phase can last several weeks after implantation. Suboptimal cement technique and application can lead to aseptic loosening, which is one of the main causes of implant failure [9]. Therefore, exploring suitable conditions to reduce the implant loosening rate is necessary. We considered a new angle by changing the temperature of the prosthesis to see if there is an impact on the cement fixation strength. In this experiment, femoral prosthesis and tibial plateau prosthesis with different temperature gradients were designed and then bonded with polypropylene resin base with bone cement to simulate intraoperative bonding, and the fixation strength of bone cement was evaluated by measuring the push-out force of the model after solidification.

By observing the effect of prosthesis temperature on the strength and porosity of bone cement fixation, and to evaluate the feasibility and effectiveness of this method for reducing the rate of prosthesis loosening after TKA, and providing the scientific basis and operational guidance for clinical surgery.

## Materials and methods

**Fig. 1:**
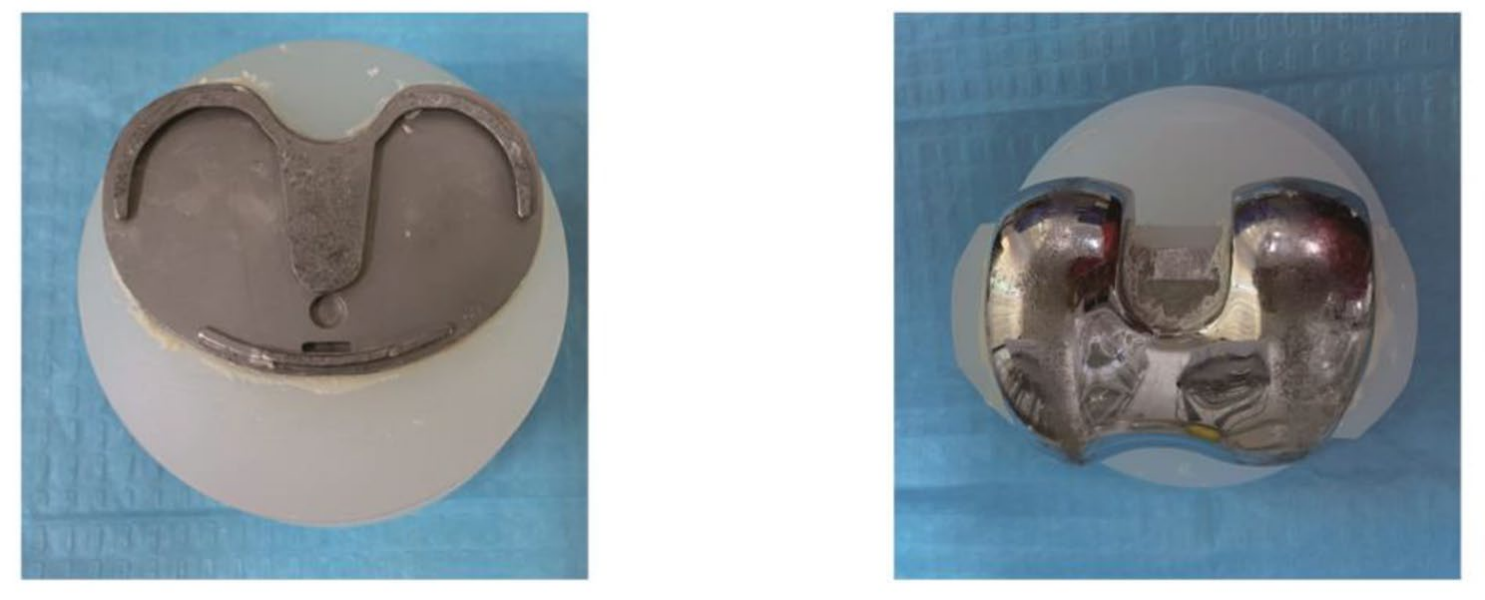
Fixed knee prosthesis

Experimental materials: The launch test uses a universal electronic mechanics machine (Shenzhen Wance Co., Ltd., ETM104B) to measure the bonding force between the metal prosthesis and the joint model made of the base after the bone cement is cured. The femoral prosthesis is size 2, and the tibial plateau prosthesis is size 2+ (Wright Medical Technology Group, USA). The tibial plateau prosthesis and the femoral prosthesis are used for 3D modeling(Autodesk 3ds Max). The modeling data was cast into a cylindrical femoral prosthesis base (9 cm in diameter, 6.4 cm in height) and a tibial plateau prosthesis base (8 cm in diameter, 6.4 cm in height), which were made of polypropylene cylinders (Qingdao Guoke Hongcheng Radio and Television Technology Co., Ltd.) (Fig. 1), the femoral prosthesis base must be processed with a 17 mm diameter through-center hole in the center of the cylinder, and the tibial prosthesis have an angle of 135°between the two wings except for the 17 mm-diameter through-center holes. The 5 mm slit allows for a perfect fit with the keel of the tibial prosthesis platform, and the through-core hole can be used for push-out experiments with push rods. Since the base material is polypropylene, it is not affected by temperature fluctuations caused by the temperature of the prosthesis and the polymerization of the bone cement. The viscosity of polypropylene and bone cement is lower than that of bone cement and prosthesis, so the base surface should be separated from the bone cement first when performing the push-out experiment. The prosthesis was pre-cooled or pre-heated in a constant temperature shaker (Honour Tianjin Co., Ltd., HNYC-202T) according to the experimental requirements before the experiment, and they were kept at a constant temperature in the shaker during curing. The polypropylene base and the prosthesis were bonded with acrylic resin bone cement (containing antibiotics) (Biomet, France). The polypropylene base with a cylindrical alloy sleeve to facilitate the push-out experiment. The specifications are 8 cm in inner diameter, 11 cm in outer diameter, and 9 cm in height. The capacity can accommodate the joint model while leaving a space of 3 cm to facilitate the push-out experiment. Sufficient space ensures that the prosthesis and base can be easily pushed apart.

### *Method*

Femoral prostheses and tibial plateau prostheses (Table 1) were pre-cooled or pre-warmed at different temperatures (4 °C, 15 °C, 25 °C, 37 °C, 45 °C), with the same temperature of bone mixed cement, the same time of use of bone cement, and the same curing temperature were used for experimental operation.

**Table 1:** Experimental grouping of femoral and tibial plateau prosthesis

Femoral and Tibial plateau prosthesis temperature	Cases/n	Mixing Temperature(℃)	Curing Temperature(℃)	Bone cement application period
4℃	3	22℃	37℃	Dough period
15℃	3	22℃	37℃	Dough period
25℃	3	22℃	37℃	Dough period
37℃	3	22℃	37℃	Dough period
45℃	3	22℃	37℃	Dough period

**Fig. 2:**
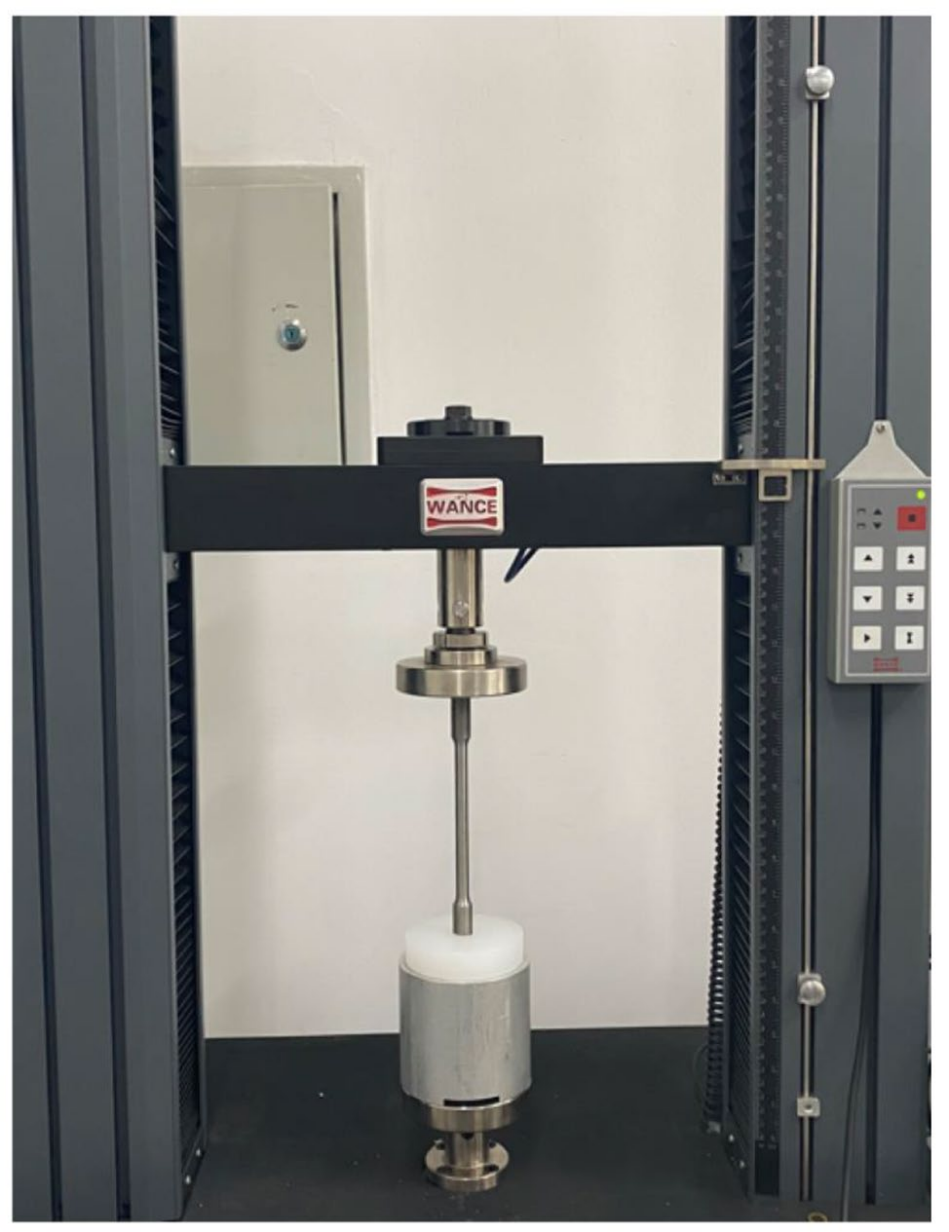
Rollout experiment in progress

**Fig. 3:**
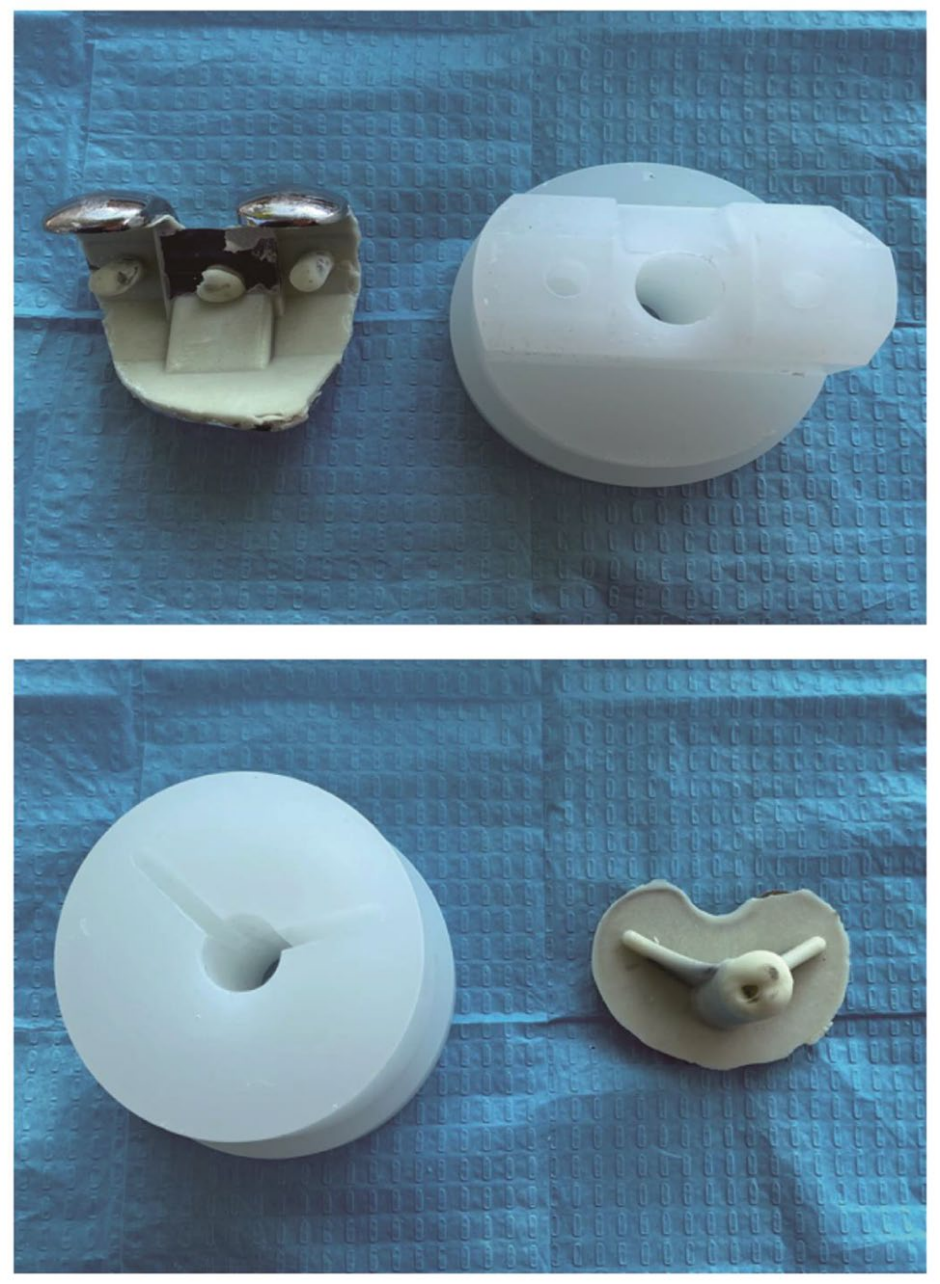
The joint prosthesis after being pushed open

Experimental operation: The entire process was completed by the same trained surgeon, and the operation process of each group was standardized, and other experimental conditions except the temperature conditions of the prosthes were the same to reduce the influence of confounding factors on the results. Before the experiment, the femoral prosthesis and the tibial plateau prosthesis were pre-cooled or pre-heated for two hours at different temperatures in a constant temperature shaking box at 4 °C, 15 °C, 25 °C, 37 °C, and 45 °C, respectively. 40.8 g of powder and 20 ml of liquid, at room temperature 22 °C and 55% air humidity, adding 40.8 g of bone cement powder into a plastic mixing bowl and then adding 20 ml of liquid monomer. Follow the operation plan of the bone cement instruction manual. After mixing, stir the cement to a homogeneous body, and the stirring time is about 30s. After stirring and waiting for the bone cement to enter the dough stage (the application period specified in the instructions for use: the dough period is 75 to 230 s after mixing when the room temperature is 22 °C), we took the prosthesis out of the constant temperature shaking box, and then immediately cemented the prosthesis with the base model. Apply the mixed cement to the tibial plateau component (including keel), femoral component (including keel), and polypropylene base surfaces. After applying appropriate force to bond the prosthesis to the base, use a spatula to remove excess bone cement ( Fig. 1), and use the universal electronic mechanics machine to apply 100 N pressure to the femoral prosthesis for 10 min. Then set the temperature in the incubator to 37 °C for curing for 24 h, and then prepare for the push-out experiment. During the mechanical test, the assembled metal support cylinder and joint model were placed on the universal electromechanical machine tray (Fig. 2). Insert a metal rod with a length of about 25 cm and a diameter of about 16 mm from the circular hole in the middle of the polypropylene base. The push rod only contacts the prosthesis keel to prevent the force from being dispersed outside the prosthesis. The joint model is vertical to prevent experimental errors caused by a thrust in other directions. The universal electromechanical machine was set to apply a constant speed of 3 mm/min to the push rod, and the push force was continuously recorded at a sampling rate of 10 Hz throughout the test process. The fracture judgment force of the bone cement and the base was set to 50 N. When the base and cement are separated, the sound of cement breaking can be heard, and it can be seen that the push-out force curve suddenly drops, and the bone cement and base surface are separated (Fig. 3). At this time, the test is stopped, and the highest push-out force of the push-out force curve is recorded.

The bone cement was removed from the prosthesis after the roll-out experiment was done and made into pieces of a certain diameter of less than 1 cm. A layer of metal was sprayed on the surface of the sample as a conductive material, and then the sample was placed under an electron microscope (SEM) to observe the fine pore structure of the bone cement.

Statistical methods: The Pearson correlation coefficient linear regression analysis was performed on the femoral prosthesis and the tibial plateau prosthesis, respectively. A multivariate analysis of variance was used between the femoral and tibial plateau prostheses. P < 0.05 was considered statistically significant(*p＜0.05). Statistical analysis was performed using SPSS 26.0.

## Results

**Fig. 4:**
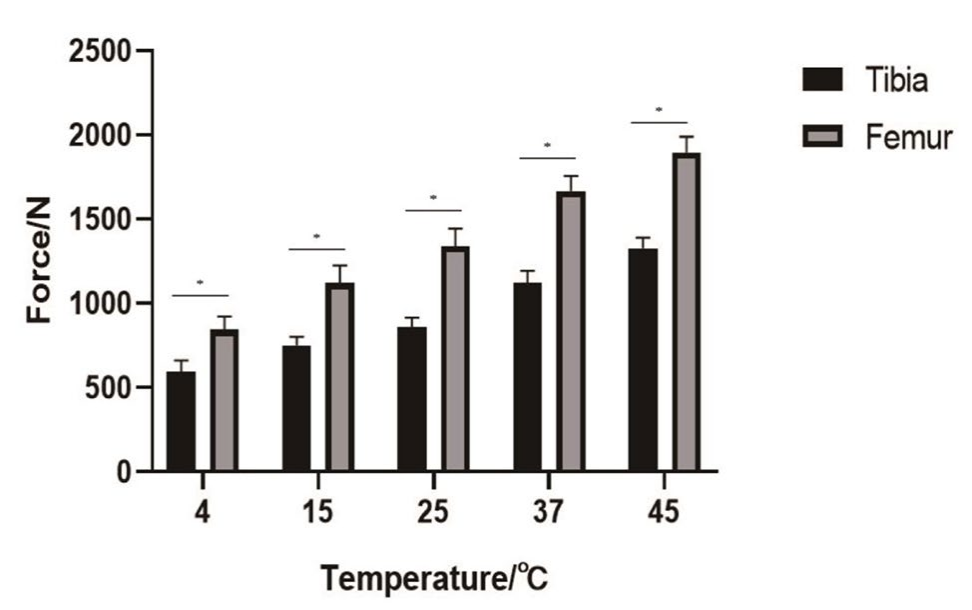
Comparison of push-out force between tibial plateau prosthesis and femoral prosthesis. Temperature = Prosthesis temperature; Force = Push-out force

**Fig. 5:**
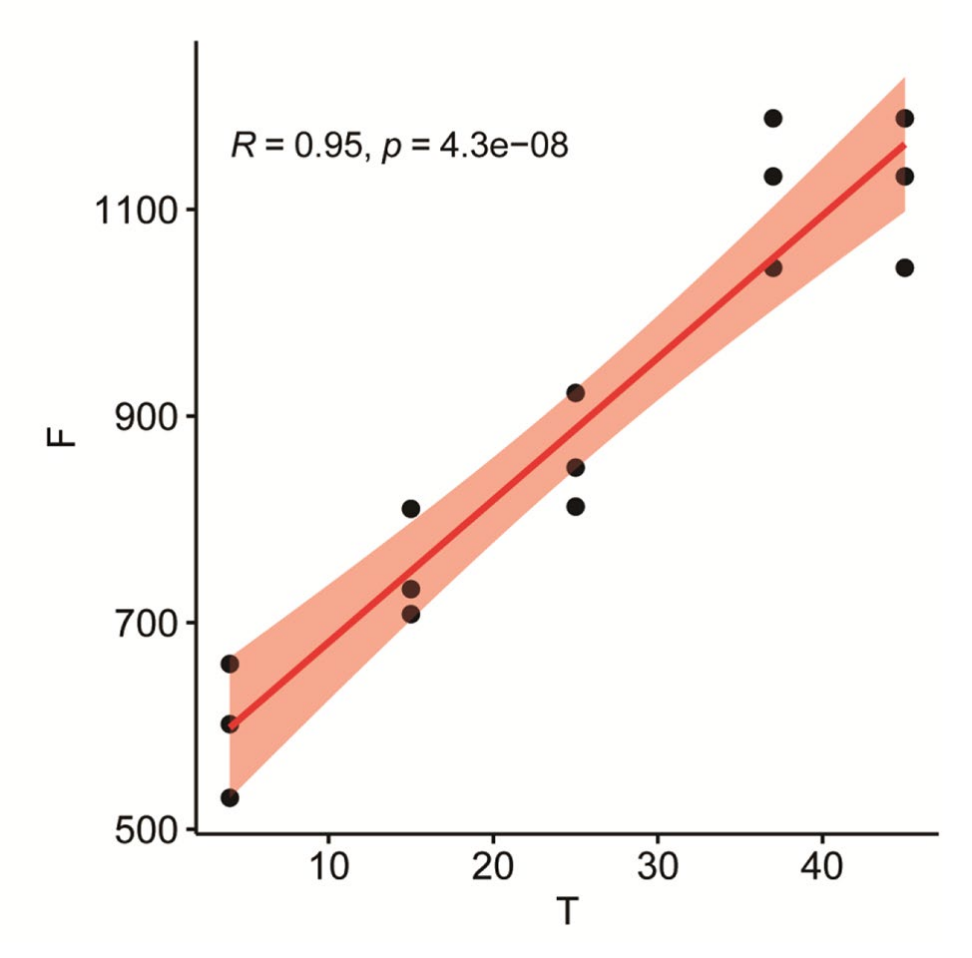
Pearson correlation coefficient analysis of tibial plateau prosthesis. T = Temperature/℃ F = Extrusion force/N

**Fig. 6:**
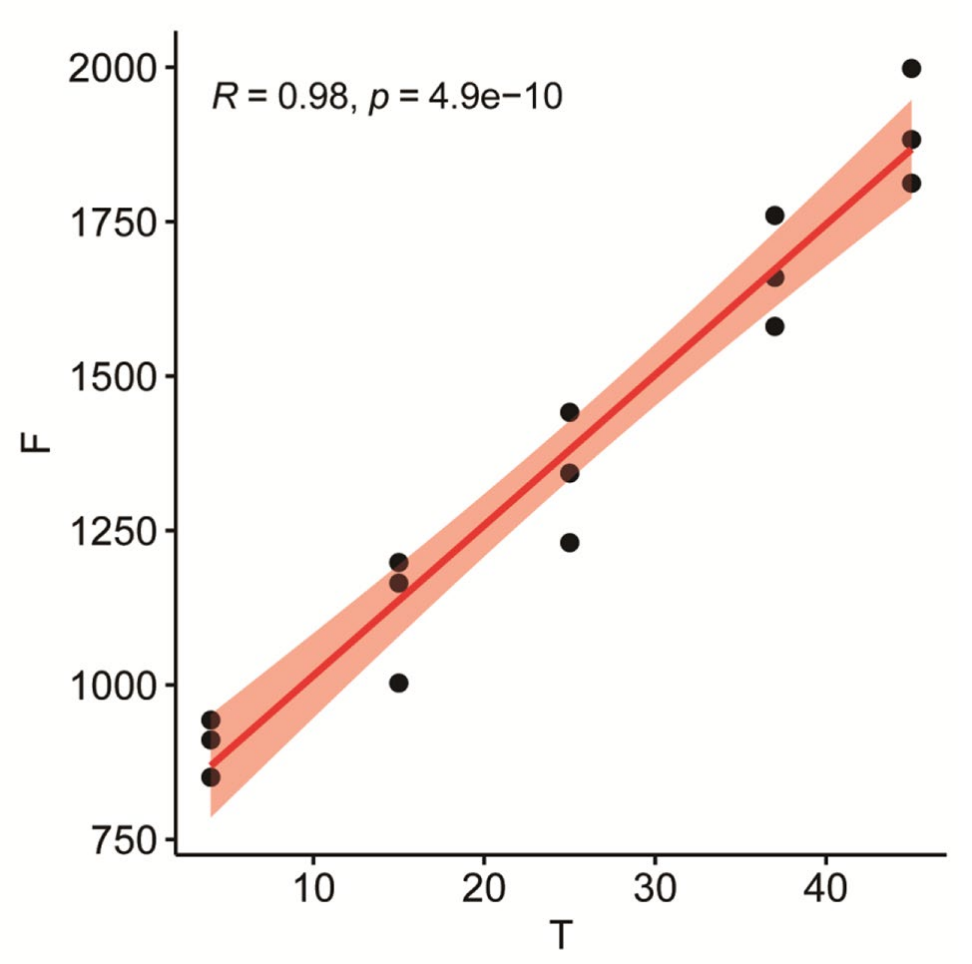
Pearson correlation coefficient analysis of femoral prosthesis. T = Temperature/℃ F = Extrusion force/N

The push-out force of the prosthesis at each temperature was: 4 °C (femur: 904.40 ± 91.97 N, tibia: 597.37 ± 64.91 N), 15 °C (femur: 1122.20 ± 104.40 N, tibia: 750.27 ± 53.30 N). 25 °C(femur: 1338.33 ± 105.58 N, tibia: 861.57 ± 55.89 N), 37 °C (femur: 1666.90 ± 90.05 N, tibia: 1121.23 ± 72.95 N), 45 °C (femur: 1897.87 ± 93.79 N, tibia: 1328.93 ± 61.16 N) (Table 2). Multivariate analysis of variance showed that the push-out force of the femoral prosthesis after fixation was higher than that of the tibial plateau prosthesis, P < 0.05 (Fig. 4). According to the Pearson correlation coefficient analysis, there was a strong correlation between the push-out force of the tibial plateau prosthesis and the prosthesis temperature (R = 0.95, P < 0.05) (Fig. 5). There was a strong correlation between the push-out force of the femoral prosthesis and the prosthesis temperature (R = 0.98, P < 0.05) (Fig. 6).

**Table 2:** The push-out force of the prosthesis at each temperature

Prosthesis Temperature/℃	Prosthesis Type	Push-out Force/N	Average Push Out Force/N
4	Tibial plateau prosthesis	601.7	597.37
		530.4	
		660	
	Femoral prosthesis	850.7	901.70
		911.2	
		943.2	
15	Tibial plateau prosthesis	732.4	750.27
		708.2	
		810.2	
	Femoral prosthesis	1165	1122.20
		1003.2	
		1198.4	
25	Tibial plateau prosthesis	850.1	861.57
		812.3	
		922.3	
	Femoral prosthesis	1343	1338.33
		1230.5	
		1441.5	
37	Tibial plateau prosthesis	1132	1121.23
		1043.5	
		1188.2	
	Femoral prosthesis	1660	1666.90
		1580.5	
		1760.2	
45	Tibial plateau prosthesis	1332	1328.93
		1266.3	
		1388.5	
	Femoral prosthesis	1883	1897.87
		1812.4	
		1998.2	

**Fig. 7:**
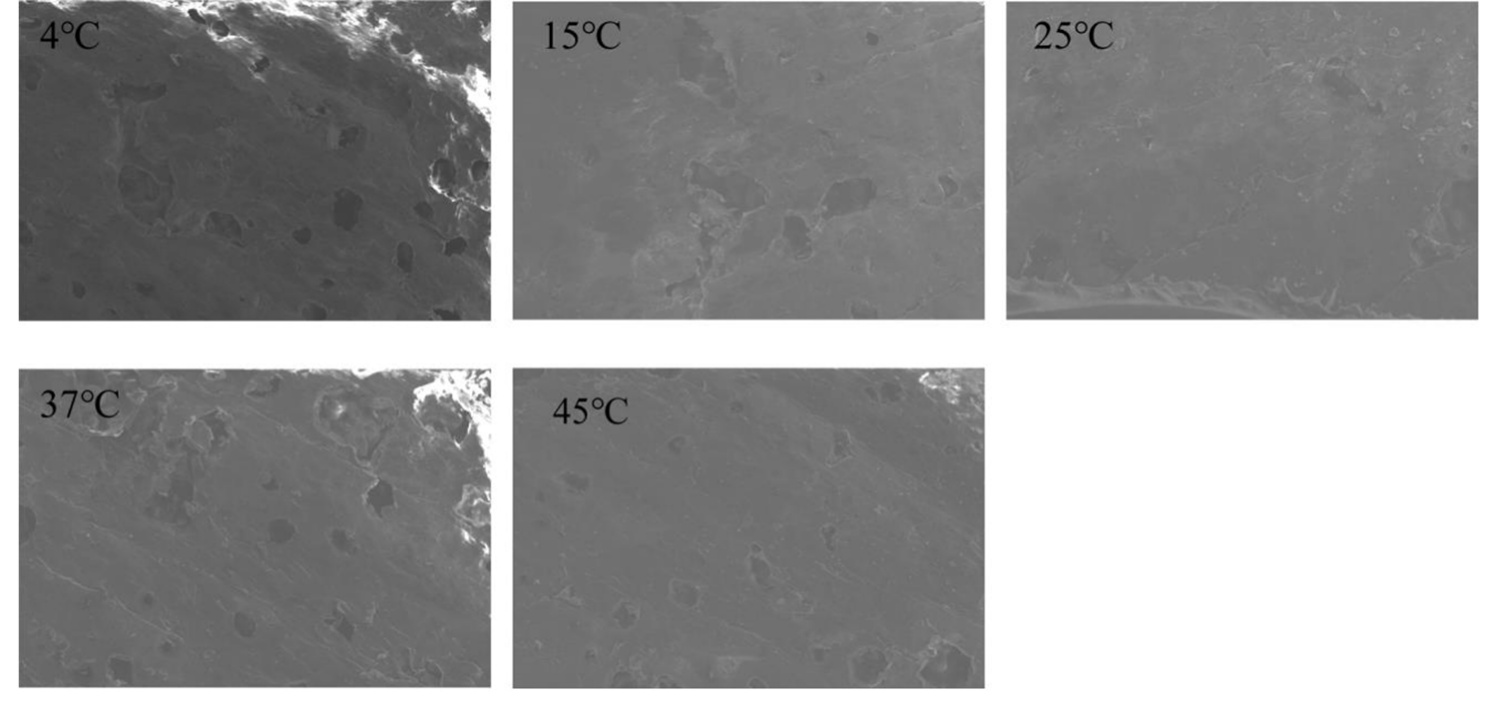
Electron microscopic morphology of the cemented pores of the tibial prosthesis

**Fig. 8:**
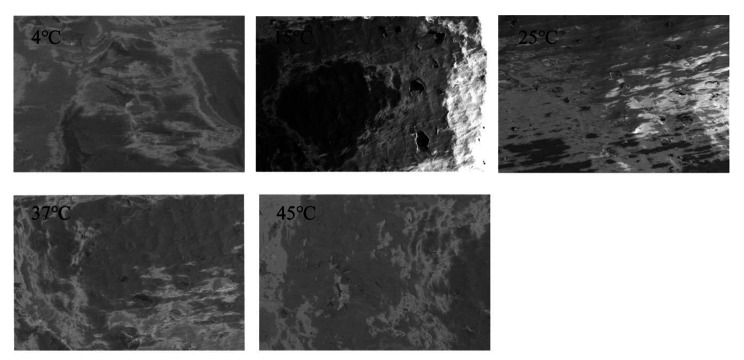
Electron microscopic morphology of the cemented pores of the femoral prosthesis

The porosity of the bone cement in the tibial (Fig. 7) and femoral (Fig. 8) prostheses was observed to decrease with increasing temperature, as evidenced by electron microscopy.

## Discussion

In recent years, the incidence of joint diseases has increased year by year, and the number of orthopedic implants used for joint replacement has increased year by year [10]. But inadequate cement technology can affect the prosthetic retention rate and other complications [11]. Aseptic loosening is one of the leading causes of implant failure, with approximately 18% of implant failures due to aseptic loosening [12]. Since the fixation strength of bone cement is an important reason for the aseptic loosening of the prosthesis, our results showed that the fixation strength of the femoral prosthesis is higher than that of the tibial plateau prosthesis, and increasing the temperature of the prosthesis before bonding can improve the fixation strength of the bone cement and prosthesis fixation strength is positively correlated with temperature, and the aim of this study was to provide some information that might reduce the incidence of tibial component loosening.

Polymethyl methacrylate (PMMA) cement has the advantages of excellent biocompatibility, good in situ formability, and sufficient strength, so it is widely used in bone cement joint replacement surgery and is a successful biomaterial in orthopedic surgery [13–15]. In vivo, experiments have shown that porous PMMA has a good effect on soft tissue coverage. In contrast to dense PMMA, porous PMMA creates a temporary gap that provides a platform for soft tissue regeneration and lays the foundation for the eventual repair of bone tissue, which is beneficial for postoperative recovery and complications [16]. However, long-term clinical observations show that PMMA cement also has some disadvantages. The most common complications of PMMA in orthopaedic applications are aseptic loosening, prosthesis infection, and thermal necrosis of surrounding tissues, especially aseptic loosening accounts for almost half of revision knee arthroplasty [17, 18]. The main cause of aseptic loosening is the lack of desirable biological and mechanical properties of PMMA cement, resulting in a weak PMMA-bone interface. X-ray photoelectron spectroscopy (XPS) and wettability studies have shown that PMMA aging increases the hydrophilicity of bone cement. Bettencourt et al. suggested that this phenomenon may affect the bone/biomaterial interface at the cellular level, leading to aseptic loosening of the implant [17]. This study investigated the fixation strength between PMMA cement and a polypropylene base during joint prosthesis fixation by means of an out-of-the-box experiment. We found that the fixation strength of the tibial plateau prosthesis was lower than that of the femoral prosthesis under the same conditions (P < 0.05). Because we control for other variables. Therefore, the consideration may be related to the larger amount of cement in the femoral prosthesis, the larger base area of the prosthesis, and the curvature of the femoral condyle. The specific mechanism remains to be discussed in the future. Terrier et al. calculated an optimal cement sheath thickness of 1.0-1.5 mm for the glenoid. In the same study, thicker cement sheaths were found to transfer excessive stress to the cement-bone interface [19]. The good ductility of bone cement makes us worry about whether the potential influencing factor of bone cement thickness between each group can be agreed upon, but each group was fixed with a constant force of 100 N for 10 min. Studies have shown that reducing porosity can prolong the fatigue life. Preheating the stem in the hip joint experiment reverses the direction of curing in the bone cement and reduces the generation of pores associated with the shrinkage process, thereby reducing bone cement porosity and increasing bone cement fatigue life [20, 21]. The bone cement completely covers all contact points of the prosthesis and the base and the thickness of the bone cement is basically the same. In addition, we found that increasing the temperature of the tibial plateau component and femoral component prior to fixation increased the fixation strength of bone cement (P < 0.05). Hip-related studies have shown that preheating the stem reverses the direction of aggregation and greatly reduces the formation of micropores at the stem-cement interface. The smaller the void volume in the bone cement, the better the compressive and flexural properties and the higher the strength of the bone cement [22]. This coincides with our findings, which also found that increased temperature reduces the pore space of bone cement. There are also studies showing that preheating the CoCrMo alloy rod before inserting the bone cement can improve its ultimate push-out load and surface shear strength [23], which is consistent with our findings. Therefore, we believe that high temperatures will reduce the porosity of the micro-interface of bone cement.

A common operational and controversial variable affecting the handling of PMMA bone cement during polymerization is ambient temperature. The higher the temperature during mixing, the faster the polymerization rate, and the shorter the working time and the setting time, but has no effect on the peak temperature. These effects have led to the popularity of pre-cooling high-viscosity cement to slow down polymerization. However, the problem with this treatment is the increased porosity due to prolonged working time, which eventually leads to a decrease in the strength of the PMMA bone cement [24]. Studies have shown that increased bone cement thickness is also accompanied by increased heat production, which increases the risk of thermal necrosis of the surrounding bone, and some studies using standard surgical techniques have shown temperatures > 50 °C for 1 min, considered the temperature necessary for osteonecrosis and exposure time, which may lead to prosthesis loosening [25, 26]. Furthermore, in vitro, measurements of temperatures have shown that preheating the stem results in a negligible increase in bone temperature [27]. Taking this into account, in our experimental design, the endpoint temperature in the variable was set at 45 °C. For the effect of polymerization temperature, chitosan (Cs) was used as an additive for PMMA, and Shi et al. [28] added chitosan (Cs) nanoparticles to bone cement. It has significant antibacterial activity against Staphylococcus aureus and Staphylococcus epidermidis, while these nanoparticles have no effect on the mechanical strength of bone cement, and through the complexation of Cs and PMMA, the polymerization temperature of the cement is significantly reduced, reducing the risk of osteonecrosis. However, we knew little about how to improve the fixation strength of bone cement, so we thought of a new angle and took the prosthesis temperature as the study variable, and found that increasing the temperature of tibial plateau prosthesis and femoral prosthesis fixation can improve the fixation strength of bone cement (P < 0.05).

Although this study focused on the fixation strength 24 h after bone cement fixation, the long-term effects were not described in detail. We controlled the endpoint temperature at 45 °C to avoid inducing osteonecrosis, but we did not conduct in-depth studies on whether the increase in prosthesis temperature and the temperature generated by bone cement polymerization induce osteonecrosis. Since the experiments in this study used polyethylene bases, which are smoother compared to human skeletal cancellous bone, the interaction between bone cement and cancellous bone could not be fully simulated. Looking forward to more in-depth studies and related clinical trials with larger samples. Although our study does not fully reproduce the in vivo environment around the prosthesis, it contributes to understanding the changes in the bone cement surface that modulate the patient’s response to the prosthesis. We believed that preheating the prosthesis temperature in advance before surgical placement of the prosthesis can improve the fixation strength of the implants and may reduce the occurrence of aseptic loosening.

## Conclusion

In summary, we made our own knee replacement model and demonstrated that elevating the temperature significantly increased the push-out force of the joint model while reducing the porosity of the bone cement. These results suggest that controlling j-prosthesis temperature is one of the very important factors in knee replacement surgery. Future studies can further explore how controlling temperature can improve surgical outcomes and thus lead to better patient outcomes.

## Data Availability

The datasets generated during and/or analysed during the current study are available from the corresponding author on reasonable request.
